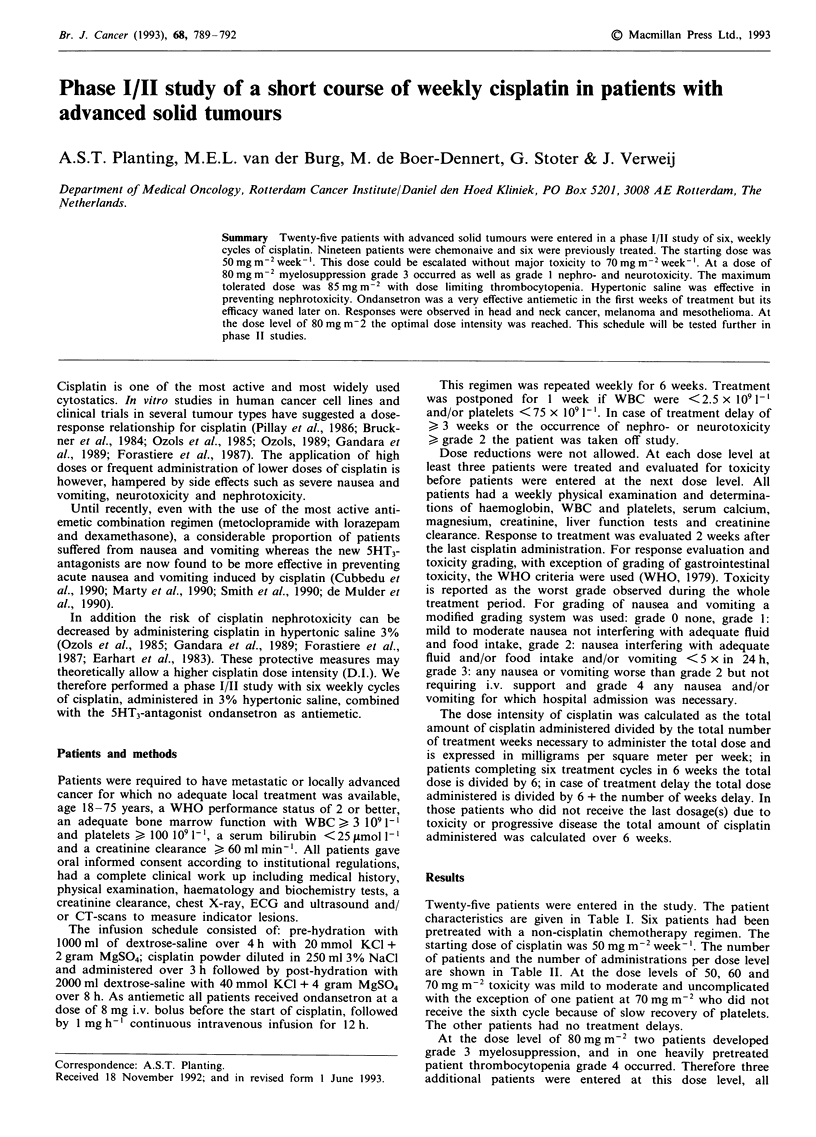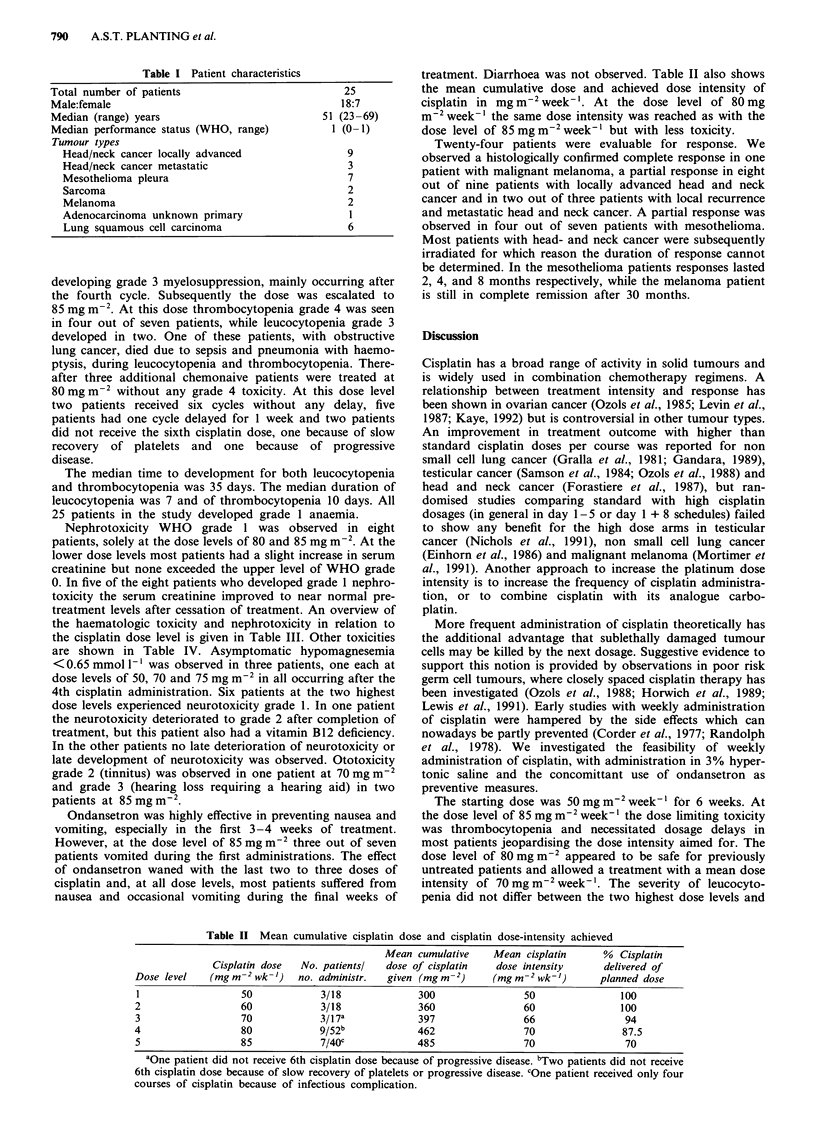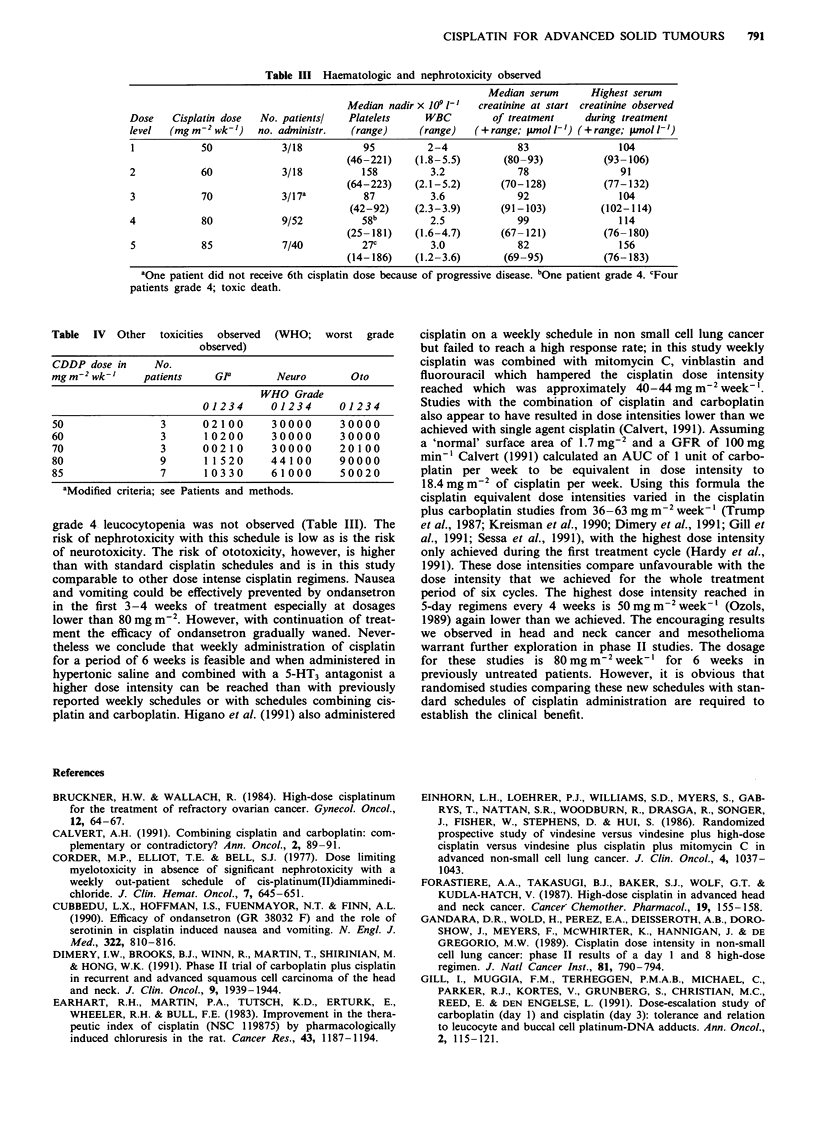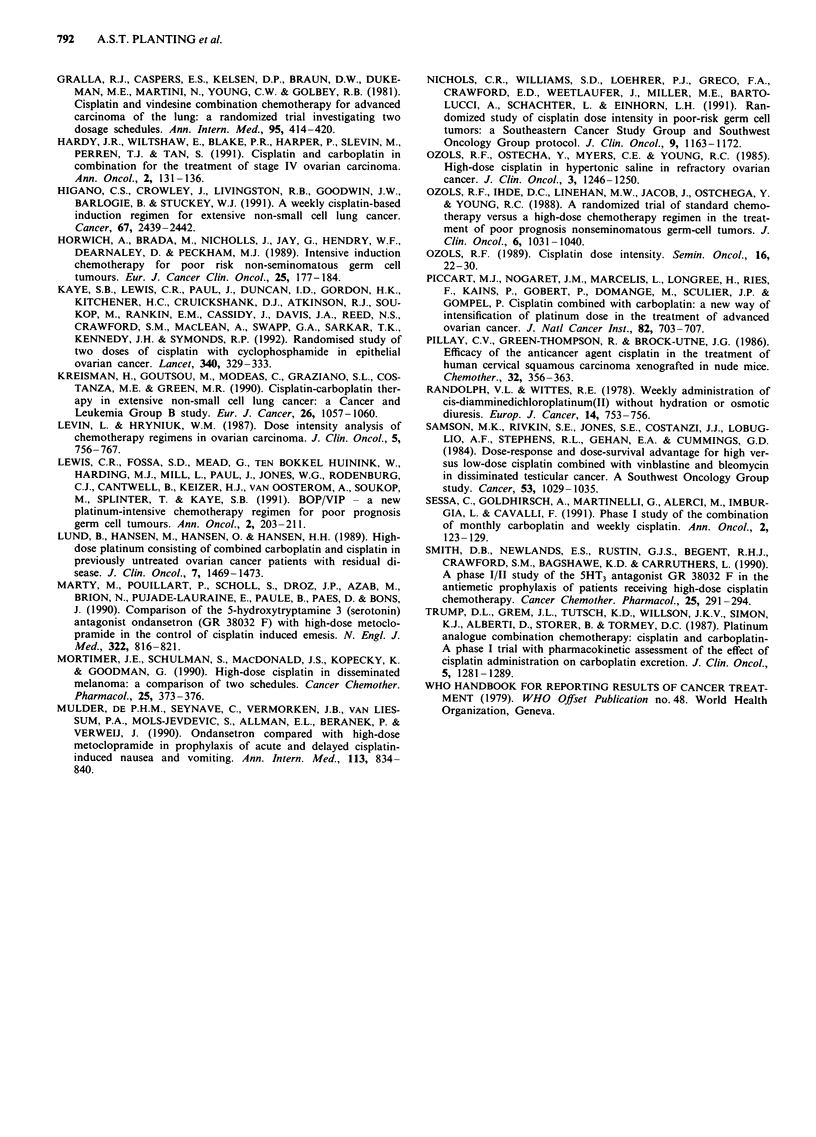# Phase I/II study of a short course of weekly cisplatin in patients with advanced solid tumours.

**DOI:** 10.1038/bjc.1993.429

**Published:** 1993-10

**Authors:** A. S. Planting, M. E. van der Burg, M. de Boer-Dennert, G. Stoter, J. Verweij

**Affiliations:** Department of Medical Oncology, Rotterdam Cancer Institute/Daniel den Hoed Kliniek, The Netherlands.

## Abstract

Twenty-five patients with advanced solid tumours were entered in a phase I/II study of six, weekly cycles of cisplatin. Nineteen patients were chemonaive and six were previously treated. The starting dose was 50 mg m-2 week-1. This dose could be escalated without major toxicity to 70 mg m-2 week-1. At a dose of 80 mg m-2 myelosuppression grade 3 occurred as well as grade 1 nephro- and neurotoxicity. The maximum tolerated dose was 85 mg m-2 with dose limiting thrombocytopenia. Hypertonic saline was effective in preventing nephrotoxicity. Ondansetron was a very effective antiemetic in the first weeks of treatment but its efficacy waned later on. Responses were observed in head and neck cancer, melanoma and mesothelioma. At the dose level of 80 mg m-2 the optimal dose intensity was reached. This schedule will be tested further in phase II studies.


*Br. J. Cancer* (1993), **68**, 789–792 © Macmillan Press Ltd., 1993

### Phase I/II study of a short course of weekly cisplatin in patients with advanced solid tumours

A.S.T. Planting, M.E.L. van der Burg, M. de Boer-Dennert, G. Stoter & J. Verweij

*Department of Medical Oncology, Rotterdam Cancer Institute/Daniel den Hoed Kliniek, PO Box 5201, 3008 AE Rotterdam, The Netherlands.*

**Summary** Twenty-five patients with advanced solid tumours were entered in a phase I/II study of six, weekly cycles of cisplatin. Nineteen patients were chemonaive and six were previously treated. The starting dose was 50 mg m$^{-2}$ week$^{-1}$. This dose could be escalated without major toxicity to 70 mg m$^{-2}$ week$^{-1}$. At a dose of 80 mg m$^{-2}$ myelosuppression grade 3 occurred as well as grade 1 nephro- and neurotoxicity. The maximum tolerated dose was 85 mg m$^{-2}$ with dose limiting thrombocytopenia. Hypertonic saline was effective in preventing nephrotoxicity. Ondansetron was a very effective antiemetic in the first weeks of treatment but its efficacy waned later on. Responses were observed in head and neck cancer, melanoma and mesothelioma. At the dose level of 80 mg m$^{-}$2 the optimal dose intensity was reached. This schedule will be tested further in phase II studies.

Cisplatin is one of the most active and most widely used cytostatics. *In vitro* studies in human cancer cell lines and clinical trials in several tumour types have suggested a dose-response relationship for cisplatin (Pillay *et al.*, 1986; Bruckner *et al.*, 1984; Ozols *et al.*, 1985; Ozols, 1989; Gandara *et al.*, 1989; Forastiere *et al.*, 1987). The application of high doses or frequent administration of lower doses of cisplatin is however, hampered by side effects such as severe nausea and vomiting, neurotoxicity and nephrotoxicity.

Until recently, even with the use of the most active anti-emetic combination regimen (metoclopramide with lorazepam and dexamethasone), a considerable proportion of patients suffered from nausea and vomiting whereas the new $5HT_3$-antagonists are now found to be more effective in preventing acute nausea and vomiting induced by cisplatin (Cubbedu *et al.*, 1990; Marty *et al.*, 1990; Smith *et al.*, 1990; de Mulder *et al.*, 1990).

In addition the risk of cisplatin nephrotoxicity can be decreased by administering cisplatin in hypertonic saline 3% (Ozols *et al.*, 1985; Gandara *et al.*, 1989; Forastiere *et al.*, 1987; Earhart *et al.*, 1983). These protective measures may theoretically allow a higher cisplatin dose intensity (D.I.). We therefore performed a phase I/II study with six weekly cycles of cisplatin, administered in 3% hypertonic saline, combined with the $5HT_3$-antagonist ondansetron as antiemetic.

### Patients and methods

Patients were required to have metastatic or locally advanced cancer for which no adequate local treatment was available, age 18–75 years, a WHO performance status of 2 or better, an adequate bone marrow function with WBC $\geqslant 3\ 10^9\ l^{-1}$ and platelets $\geqslant 100\ 10^9\ l^{-1}$, a serum bilirubin $< 25\ \mu mol\ l^{-1}$ and a creatinine clearance $\geqslant 60\ ml\ min^{-1}$. All patients gave oral informed consent according to institutional regulations, had a complete clinical work up including medical history, physical examination, haematology and biochemistry tests, a creatinine clearance, chest X-ray, ECG and ultrasound and/or CT-scans to measure indicator lesions.

The infusion schedule consisted of: pre-hydration with 1000 ml of dextrose-saline over 4 h with 20 mmol KCl + 2 gram $MgSO_4$; cisplatin powder diluted in 250 ml 3% NaCl and administered over 3 h followed by post-hydration with 2000 ml dextrose-saline with 40 mmol KCl + 4 gram $MgSO_4$ over 8 h. As antiemetic all patients received ondansetron at a dose of 8 mg i.v. bolus before the start of cisplatin, followed by 1 mg h$^{-1}$ continuous intravenous infusion for 12 h.

This regimen was repeated weekly for 6 weeks. Treatment was postponed for 1 week if WBC were $< 2.5 \times 10^9\ l^{-1}$ and/or platelets $< 75 \times 10^9\ l^{-1}$. In case of treatment delay of $\geqslant 3$ weeks or the occurrence of nephro- or neurotoxicity $\geqslant$ grade 2 the patient was taken off study.

Dose reductions were not allowed. At each dose level at least three patients were treated and evaluated for toxicity before patients were entered at the next dose level. All patients had a weekly physical examination and determinations of haemoglobin, WBC and platelets, serum calcium, magnesium, creatinine, liver function tests and creatinine clearance. Response to treatment was evaluated 2 weeks after the last cisplatin administration. For response evaluation and toxicity grading, with exception of grading of gastrointestinal toxicity, the WHO criteria were used (WHO, 1979). Toxicity is reported as the worst grade observed during the whole treatment period. For grading of nausea and vomiting a modified grading system was used: grade 0 none, grade 1: mild to moderate nausea not interfering with adequate fluid and food intake, grade 2: nausea interfering with adequate fluid and/or food intake and/or vomiting $< 5 \times$ in 24 h, grade 3: any nausea or vomiting worse than grade 2 but not requiring i.v. support and grade 4 any nausea and/or vomiting for which hospital admission was necessary.

The dose intensity of cisplatin was calculated as the total amount of cisplatin administered divided by the total number of treatment weeks necessary to administer the total dose and is expressed in milligrams per square meter per week; in patients completing six treatment cycles in 6 weeks the total dose is divided by 6; in case of treatment delay the total dose administered is divided by 6 + the number of weeks delay. In those patients who did not receive the last dosage(s) due to toxicity or progressive disease the total amount of cisplatin administered was calculated over 6 weeks.

### Results

Twenty-five patients were entered in the study. The patient characteristics are given in Table I. Six patients had been pretreated with a non-cisplatin chemotherapy regimen. The starting dose of cisplatin was 50 mg m$^{-2}$ week$^{-1}$. The number of patients and the number of administrations per dose level are shown in Table II. At the dose levels of 50, 60 and 70 mg m$^{-2}$ toxicity was mild to moderate and uncomplicated with the exception of one patient at 70 mg m$^{-2}$ who did not receive the sixth cycle because of slow recovery of platelets. The other patients had no treatment delays.

At the dose level of 80 mg m$^{-2}$ two patients developed grade 3 myelosuppression, and in one heavily pretreated patient thrombocytopenia grade 4 occurred. Therefore three additional patients were entered at this dose level, all

Correspondence: A.S.T. Planting.
Received 18 November 1992; and in revised form 1 June 1993.

**Table I** Patient characteristics

| | |
|---|---|
| Total number of patients | 25 |
| Male:female | 18:7 |
| Median (range) years | 51 (23–69) |
| Median performance status (WHO, range) | 1 (0–1) |
| *Tumour types* | |
| Head/neck cancer locally advanced | 9 |
| Head/neck cancer metastatic | 3 |
| Mesothelioma pleura | 7 |
| Sarcoma | 2 |
| Melanoma | 2 |
| Adenocarcinoma unknown primary | 1 |
| Lung squamous cell carcinoma | 6 |

developing grade 3 myelosuppression, mainly occurring after the fourth cycle. Subsequently the dose was escalated to 85 mg $m^{-2}$. At this dose thrombocytopenia grade 4 was seen in four out of seven patients, while leucocytopenia grade 3 developed in two. One of these patients, with obstructive lung cancer, died due to sepsis and pneumonia with haemoptysis, during leucocytopenia and thrombocytopenia. Thereafter three additional chemonaive patients were treated at 80 mg $m^{-2}$ without any grade 4 toxicity. At this dose level two patients received six cycles without any delay, five patients had one cycle delayed for 1 week and two patients did not receive the sixth cisplatin dose, one because of slow recovery of platelets and one because of progressive disease.

The median time to development for both leucocytopenia and thrombocytopenia was 35 days. The median duration of leucocytopenia was 7 and of thrombocytopenia 10 days. All 25 patients in the study developed grade 1 anaemia.

Nephrotoxicity WHO grade 1 was observed in eight patients, solely at the dose levels of 80 and 85 mg $m^{-2}$. At the lower dose levels most patients had a slight increase in serum creatinine but none exceeded the upper level of WHO grade 0. In five of the eight patients who developed grade 1 nephrotoxicity the serum creatinine improved to near normal pretreatment levels after cessation of treatment. An overview of the haematologic toxicity and nephrotoxicity in relation to the cisplatin dose level is given in Table III. Other toxicities are shown in Table IV. Asymptomatic hypomagnesemia $<0.65$ mmol $l^{-1}$ was observed in three patients, one each at dose levels of 50, 70 and 75 mg $m^{-2}$ in all occurring after the 4th cisplatin administration. Six patients at the two highest dose levels experienced neurotoxicity grade 1. In one patient the neurotoxicity deteriorated to grade 2 after completion of treatment, but this patient also had a vitamin B12 deficiency. In the other patients no late deterioration of neurotoxicity or late development of neurotoxicity was observed. Ototoxicity grade 2 (tinnitus) was observed in one patient at 70 mg $m^{-2}$ and grade 3 (hearing loss requiring a hearing aid) in two patients at 85 mg $m^{-2}$.

Ondansetron was highly effective in preventing nausea and vomiting, especially in the first 3–4 weeks of treatment. However, at the dose level of 85 mg $m^{-2}$ three out of seven patients vomited during the first administrations. The effect of ondansetron waned with the last two to three doses of cisplatin and, at all dose levels, most patients suffered from nausea and occasional vomiting during the final weeks of treatment. Diarrhoea was not observed. Table II also shows the mean cumulative dose and achieved dose intensity of cisplatin in mg $m^{-2}$ $week^{-1}$. At the dose level of 80 mg $m^{-2}$ $week^{-1}$ the same dose intensity was reached as with the dose level of 85 mg $m^{-2}$ $week^{-1}$ but with less toxicity.

Twenty-four patients were evaluable for response. We observed a histologically confirmed complete response in one patient with malignant melanoma, a partial response in eight out of nine patients with locally advanced head and neck cancer and in two out of three patients with local recurrence and metastatic head and neck cancer. A partial response was observed in four out of seven patients with mesothelioma. Most patients with head- and neck cancer were subsequently irradiated for which reason the duration of response cannot be determined. In the mesothelioma patients responses lasted 2, 4, and 8 months respectively, while the melanoma patient is still in complete remission after 30 months.

### Discussion

Cisplatin has a broad range of activity in solid tumours and is widely used in combination chemotherapy regimens. A relationship between treatment intensity and response has been shown in ovarian cancer (Ozols *et al.*, 1985; Levin *et al.*, 1987; Kaye, 1992) but is controversial in other tumour types. An improvement in treatment outcome with higher than standard cisplatin doses per course was reported for non small cell lung cancer (Gralla *et al.*, 1981; Gandara, 1989), testicular cancer (Samson *et al.*, 1984; Ozols *et al.*, 1988) and head and neck cancer (Forastiere *et al.*, 1987), but randomised studies comparing standard with high cisplatin dosages (in general in day 1–5 or day 1 + 8 schedules) failed to show any benefit for the high dose arms in testicular cancer (Nichols *et al.*, 1991), non small cell lung cancer (Einhorn *et al.*, 1986) and malignant melanoma (Mortimer *et al.*, 1991). Another approach to increase the platinum dose intensity is to increase the frequency of cisplatin administration, or to combine cisplatin with its analogue carboplatin.

More frequent administration of cisplatin theoretically has the additional advantage that sublethally damaged tumour cells may be killed by the next dosage. Suggestive evidence to support this notion is provided by observations in poor risk germ cell tumours, where closely spaced cisplatin therapy has been investigated (Ozols *et al.*, 1988; Horwich *et al.*, 1989; Lewis *et al.*, 1991). Early studies with weekly administration of cisplatin were hampered by the side effects which can nowadays be partly prevented (Corder *et al.*, 1977; Randolph *et al.*, 1978). We investigated the feasibility of weekly administration of cisplatin, with administration in 3% hypertonic saline and the concomittant use of ondansetron as preventive measures.

The starting dose was 50 mg $m^{-2}$ $week^{-1}$ for 6 weeks. At the dose level of 85 mg $m^{-2}$ $week^{-1}$ the dose limiting toxicity was thrombocytopenia and necessitated dosage delays in most patients jeopardising the dose intensity aimed for. The dose level of 80 mg $m^{-2}$ appeared to be safe for previously untreated patients and allowed a treatment with a mean dose intensity of 70 mg $m^{-2}$ $week^{-1}$. The severity of leucocytopenia did not differ between the two highest dose levels and

**Table II** Mean cumulative cisplatin dose and cisplatin dose-intensity achieved

| Dose level | *Cisplatin dose ($mg\ m^{-2}\ wk^{-1}$)* | *No. patients/ no. administr.* | *Mean cumulative dose of cisplatin given ($mg\ m^{-2}$)* | *Mean cisplatin dose intensity ($mg\ m^{-2}\ wk^{-1}$)* | *% Cisplatin delivered of planned dose* |
|---|---|---|---|---|---|
| 1 | 50 | 3/18 | 300 | 50 | 100 |
| 2 | 60 | 3/18 | 360 | 60 | 100 |
| 3 | 70 | 3/17[a] | 397 | 66 | 94 |
| 4 | 80 | 9/52[b] | 462 | 70 | 87.5 |
| 5 | 85 | 7/40[c] | 485 | 70 | 70 |

[a]One patient did not receive 6th cisplatin dose because of progressive disease. [b]Two patients did not receive 6th cisplatin dose because of slow recovery of platelets or progressive disease. [c]One patient received only four courses of cisplatin because of infectious complication.

**Table III** Haematologic and nephrotoxicity observed

| Dose level | Cisplatin dose ($mg\,m^{-2}\,wk^{-1}$) | No. patients/ no. administr. | Median nadir $\times 10^9\,l^{-1}$ Platelets (range) | WBC (range) | Median serum creatinine at start of treatment (+range; $\mu mol\,l^{-1}$) | Highest serum creatinine observed during treatment (+range; $\mu mol\,l^{-1}$) |
|---|---|---|---|---|---|---|
| 1 | 50 | 3/18 | 95 (46–221) | 2–4 (1.8–5.5) | 83 (80–93) | 104 (93–106) |
| 2 | 60 | 3/18 | 158 (64–223) | 3.2 (2.1–5.2) | 78 (70–128) | 91 (77–132) |
| 3 | 70 | 3/17[a] | 87 (42–92) | 3.6 (2.3–3.9) | 92 (91–103) | 104 (102–114) |
| 4 | 80 | 9/52 | 58[b] (25–181) | 2.5 (1.6–4.7) | 99 (67–121) | 114 (76–180) |
| 5 | 85 | 7/40 | 27[c] (14–186) | 3.0 (1.2–3.6) | 82 (69–95) | 156 (76–183) |

[a]One patient did not receive 6th cisplatin dose because of progressive disease. [b]One patient grade 4. [c]Four patients grade 4; toxic death.

**Table IV** Other toxicities observed (WHO; worst grade observed)

| CDDP dose in $mg\,m^{-2}\,wk^{-1}$ | No. patients | GI[a] | Neuro | Oto |
|---|---|---|---|---|
| | | | WHO Grade | |
| | | 0 1 2 3 4 | 0 1 2 3 4 | 0 1 2 3 4 |
| 50 | 3 | 0 2 1 0 0 | 3 0 0 0 0 | 3 0 0 0 0 |
| 60 | 3 | 1 0 2 0 0 | 3 0 0 0 0 | 3 0 0 0 0 |
| 70 | 3 | 0 0 2 1 0 | 3 0 0 0 0 | 2 0 1 0 0 |
| 80 | 9 | 1 1 5 2 0 | 4 4 1 0 0 | 9 0 0 0 0 |
| 85 | 7 | 1 0 3 3 0 | 6 1 0 0 0 | 5 0 0 2 0 |

[a]Modified criteria; see Patients and methods.

grade 4 leucocytopenia was not observed (Table III). The risk of nephrotoxicity with this schedule is low as is the risk of neurotoxicity. The risk of ototoxicity, however, is higher than with standard cisplatin schedules and is in this study comparable to other dose intense cisplatin regimens. Nausea and vomiting could be effectively prevented by ondansetron in the first 3–4 weeks of treatment especially at dosages lower than $80\,mg\,m^{-2}$. However, with continuation of treatment the efficacy of ondansetron gradually waned. Nevertheless we conclude that weekly administration of cisplatin for a period of 6 weeks is feasible and when administered in hypertonic saline and combined with a 5-$HT_3$ antagonist a higher dose intensity can be reached than with previously reported weekly schedules or with schedules combining cisplatin and carboplatin. Higano *et al.* (1991) also administered cisplatin on a weekly schedule in non small cell lung cancer but failed to reach a high response rate; in this study weekly cisplatin was combined with mitomycin C, vinblastin and fluorouracil which hampered the cisplatin dose intensity reached which was approximately $40–44\,mg\,m^{-2}\,week^{-1}$. Studies with the combination of cisplatin and carboplatin also appear to have resulted in dose intensities lower than we achieved with single agent cisplatin (Calvert, 1991). Assuming a 'normal' surface area of $1.7\,mg^{-2}$ and a GFR of 100 mg $min^{-1}$ Calvert (1991) calculated an AUC of 1 unit of carboplatin per week to be equivalent in dose intensity to $18.4\,mg\,m^{-2}$ of cisplatin per week. Using this formula the cisplatin equivalent dose intensities varied in the cisplatin plus carboplatin studies from $36–63\,mg\,m^{-2}\,week^{-1}$ (Trump *et al.*, 1987; Kreisman *et al.*, 1990; Dimery *et al.*, 1991; Gill *et al.*, 1991; Sessa *et al.*, 1991), with the highest dose intensity only achieved during the first treatment cycle (Hardy *et al.*, 1991). These dose intensities compare unfavourable with the dose intensity that we achieved for the whole treatment period of six cycles. The highest dose intensity reached in 5-day regimens every 4 weeks is $50\,mg\,m^{-2}\,week^{-1}$ (Ozols, 1989) again lower than we achieved. The encouraging results we observed in head and neck cancer and mesothelioma warrant further exploration in phase II studies. The dosage for these studies is $80\,mg\,m^{-2}\,week^{-1}$ for 6 weeks in previously untreated patients. However, it is obvious that randomised studies comparing these new schedules with standard schedules of cisplatin administration are required to establish the clinical benefit.

### References

BRUCKNER, H.W. & WALLACH, R. (1984). High-dose cisplatinum for the treatment of refractory ovarian cancer. *Gynecol. Oncol.*, **12,** 64–67.

CALVERT, A.H. (1991). Combining cisplatin and carboplatin: complementary or contradictory? *Ann. Oncol.*, **2,** 89–91.

CORDER, M.P., ELLIOT, T.E. & BELL, S.J. (1977). Dose limiting myelotoxicity in absence of significant nephrotoxicity with a weekly out-patient schedule of cis-platinum(II)diamminedichloride. *J. Clin. Hemat. Oncol.*, **7,** 645–651.

CUBBEDU, L.X., HOFFMAN, I.S., FUENMAYOR, N.T. & FINN, A.L. (1990). Efficacy of ondansetron (GR 38032 F) and the role of serotinin in cisplatin induced nausea and vomiting. *N. Engl. J. Med.*, **322,** 810–816.

DIMERY, I.W., BROOKS, B.J., WINN, R., MARTIN, T., SHIRINIAN, M. & HONG, W.K. (1991). Phase II trial of carboplatin plus cisplatin in recurrent and advanced squamous cell carcinoma of the head and neck. *J. Clin. Oncol.*, **9,** 1939–1944.

EARHART, R.H., MARTIN, P.A., TUTSCH, K.D., ERTURK, E., WHEELER, R.H. & BULL, F.E. (1983). Improvement in the therapeutic index of cisplatin (NSC 119875) by pharmacologically induced chloruresis in the rat. *Cancer Res.*, **43,** 1187–1194.

EINHORN, L.H., LOEHRER, P.J., WILLIAMS, S.D., MYERS, S., GABRYS, T., NATTAN, S.R., WOODBURN, R., DRASGA, R., SONGER, J., FISHER, W., STEPHENS, D. & HUI, S. (1986). Randomized prospective study of vindesine versus vindesine plus high-dose cisplatin versus vindesine plus cisplatin plus mitomycin C in advanced non-small cell lung cancer. *J. Clin. Oncol.*, **4,** 1037–1043.

FORASTIERE, A.A., TAKASUGI, B.J., BAKER, S.J., WOLF, G.T. & KUDLA-HATCH, V. (1987). High-dose cisplatin in advanced head and neck cancer. *Cancer Chemother. Pharmacol.*, **19,** 155–158.

GANDARA, D.R., WOLD, H., PEREZ, E.A., DEISSEROTH, A.B., DOROSHOW, J., MEYERS, F., MCWHIRTER, K., HANNIGAN, J. & DE GREGORIO, M.W. (1989). Cisplatin dose intensity in non-small cell lung cancer: phase II results of a day 1 and 8 high-dose regimen. *J. Natl Cancer Inst.*, **81,** 790–794.

GILL, I., MUGGIA, F.M., TERHEGGEN, P.M.A.B., MICHAEL, C., PARKER, R.J., KORTES, V., GRUNBERG, S., CHRISTIAN, M.C., REED, E. & DEN ENGELSE, L. (1991). Dose-escalation study of carboplatin (day 1) and cisplatin (day 3): tolerance and relation to leucocyte and buccal cell platinum-DNA adducts. *Ann. Oncol.*, **2,** 115–121.

GRALLA, R.J., CASPERS, E.S., KELSEN, D.P., BRAUN, D.W., DUKEMAN, M.E., MARTINI, N., YOUNG, C.W. & GOLBEY, R.B. (1981). Cisplatin and vindesine combination chemotherapy for advanced carcinoma of the lung: a randomized trial investigating two dosage schedules. *Ann. Intern. Med.*, **95**, 414–420.

HARDY, J.R., WILTSHAW, E., BLAKE, P.R., HARPER, P., SLEVIN, M., PERREN, T.J. & TAN, S. (1991). Cisplatin and carboplatin in combination for the treatment of stage IV ovarian carcinoma. *Ann. Oncol.*, **2**, 131–136.

HIGANO, C.S., CROWLEY, J., LIVINGSTON, R.B., GOODWIN, J.W., BARLOGIE, B. & STUCKEY, W.J. (1991). A weekly cisplatin-based induction regimen for extensive non-small cell lung cancer. *Cancer*, **67**, 2439–2442.

HORWICH, A., BRADA, M., NICHOLLS, J., JAY, G., HENDRY, W.F., DEARNALEY, D. & PECKHAM, M.J. (1989). Intensive induction chemotherapy for poor risk non-seminomatous germ cell tumours. *Eur. J. Cancer Clin. Oncol.*, **25**, 177–184.

KAYE, S.B., LEWIS, C.R., PAUL, J., DUNCAN, I.D., GORDON, H.K., KITCHENER, H.C., CRUICKSHANK, D.J., ATKINSON, R.J., SOUKOP, M., RANKIN, E.M., CASSIDY, J., DAVIS, J.A., REED, N.S., CRAWFORD, S.M., MACLEAN, A., SWAPP, G.A., SARKAR, T.K., KENNEDY, J.H. & SYMONDS, R.P. (1992). Randomised study of two doses of cisplatin with cyclophosphamide in epithelial ovarian cancer. *Lancet*, **340**, 329–333.

KREISMAN, H., GOUTSOU, M., MODEAS, C., GRAZIANO, S.L., COSTANZA, M.E. & GREEN, M.R. (1990). Cisplatin-carboplatin therapy in extensive non-small cell lung cancer: a Cancer and Leukemia Group B study. *Eur. J. Cancer*, **26**, 1057–1060.

LEVIN, L. & HRYNIUK, W.M. (1987). Dose intensity analysis of chemotherapy regimens in ovarian carcinoma. *J. Clin. Oncol.*, **5**, 756–767.

LEWIS, C.R., FOSSA, S.D., MEAD, G., TEN BOKKEL HUININK, W., HARDING, M.J., MILL, L., PAUL, J., JONES, W.G., RODENBURG, C.J., CANTWELL, B., KEIZER, H.J., VAN OOSTEROM, A., SOUKOP, M., SPLINTER, T. & KAYE, S.B. (1991). BOP/VIP – a new platinum-intensive chemotherapy regimen for poor prognosis germ cell tumours. *Ann. Oncol.*, **2**, 203–211.

LUND, B., HANSEN, M., HANSEN, O. & HANSEN, H.H. (1989). High-dose platinum consisting of combined carboplatin and cisplatin in previously untreated ovarian cancer patients with residual disease. *J. Clin. Oncol.*, **7**, 1469–1473.

MARTY, M., POUILLART, P., SCHOLL, S., DROZ, J.P., AZAB, M., BRION, N., PUJADE-LAURAINE, E., PAULE, B., PAES, D. & BONS, J. (1990). Comparison of the 5-hydroxytryptamine 3 (serotonin) antagonist ondansetron (GR 38032 F) with high-dose metoclopramide in the control of cisplatin induced emesis. *N. Engl. J. Med.*, **322**, 816–821.

MORTIMER, J.E., SCHULMAN, S., MACDONALD, J.S., KOPECKY, K. & GOODMAN, G. (1990). High-dose cisplatin in disseminated melanoma: a comparison of two schedules. *Cancer Chemother. Pharmacol.*, **25**, 373–376.

MULDER, DE P.H.M., SEYNAVE, C., VERMORKEN, J.B., VAN LIESSUM, P.A., MOLS-JEVDEVIC, S., ALLMAN, E.L., BERANEK, P. & VERWEIJ, J. (1990). Ondansetron compared with high-dose metoclopramide in prophylaxis of acute and delayed cisplatin-induced nausea and vomiting. *Ann. Intern. Med.*, **113**, 834–840.

NICHOLS, C.R., WILLIAMS, S.D., LOEHRER, P.J., GRECO, F.A., CRAWFORD, E.D., WEETLAUFER, J., MILLER, M.E., BARTOLUCCI, A., SCHACHTER, L. & EINHORN, L.H. (1991). Randomized study of cisplatin dose intensity in poor-risk germ cell tumors: a Southeastern Cancer Study Group and Southwest Oncology Group protocol. *J. Clin. Oncol.*, **9**, 1163–1172.

OZOLS, R.F., OSTECHA, Y., MYERS, C.E. & YOUNG, R.C. (1985). High-dose cisplatin in hypertonic saline in refractory ovarian cancer. *J. Clin. Oncol.*, **3**, 1246–1250.

OZOLS, R.F., IHDE, D.C., LINEHAN, M.W., JACOB, J., OSTCHEGA, Y. & YOUNG, R.C. (1988). A randomized trial of standard chemotherapy versus a high-dose chemotherapy regimen in the treatment of poor prognosis nonseminomatous germ-cell tumors. *J. Clin. Oncol.*, **6**, 1031–1040.

OZOLS, R.F. (1989). Cisplatin dose intensity. *Semin. Oncol.*, **16**, 22–30.

PICCART, M.J., NOGARET, J.M., MARCELIS, L., LONGREE, H., RIES, F., KAINS, P., GOBERT, P., DOMANGE, M., SCULIER, J.P. & GOMPEL, P. Cisplatin combined with carboplatin: a new way of intensification of platinum dose in the treatment of advanced ovarian cancer. *J. Natl Cancer Inst.*, **82**, 703–707.

PILLAY, C.V., GREEN-THOMPSON, R. & BROCK-UTNE, J.G. (1986). Efficacy of the anticancer agent cisplatin in the treatment of human cervical squamous carcinoma xenografted in nude mice. *Chemother.*, **32**, 356–363.

RANDOLPH, V.L. & WITTES, R.E. (1978). Weekly administration of cis-diamminedichloroplatinum(II) without hydration or osmotic diuresis. *Europ. J. Cancer*, **14**, 753–756.

SAMSON, M.K., RIVKIN, S.E., JONES, S.E., COSTANZI, J.J., LOBUGLIO, A.F., STEPHENS, R.L., GEHAN, E.A. & CUMMINGS, G.D. (1984). Dose-response and dose-survival advantage for high versus low-dose cisplatin combined with vinblastine and bleomycin in disseminated testicular cancer. A Southwest Oncology Group study. *Cancer*, **53**, 1029–1035.

SESSA, C., GOLDHIRSCH, A., MARTINELLI, G., ALERCI, M., IMBURGIA, L. & CAVALLI, F. (1991). Phase I study of the combination of monthly carboplatin and weekly cisplatin. *Ann. Oncol.*, **2**, 123–129.

SMITH, D.B., NEWLANDS, E.S., RUSTIN, G.J.S., BEGENT, R.H.J., CRAWFORD, S.M., BAGSHAWE, K.D. & CARRUTHERS, L. (1990). A phase I/II study of the $5HT_3$ antagonist GR 38032 F in the antiemetic prophylaxis of patients receiving high-dose cisplatin chemotherapy. *Cancer Chemother. Pharmacol.*, **25**, 291–294.

TRUMP, D.L., GREM, J.L., TUTSCH, K.D., WILLSON, J.K.V., SIMON, K.J., ALBERTI, D., STORER, B. & TORMEY, D.C. (1987). Platinum analogue combination chemotherapy: cisplatin and carboplatin-A phase I trial with pharmacokinetic assessment of the effect of cisplatin administration on carboplatin excretion. *J. Clin. Oncol.*, **5**, 1281–1289.

WHO HANDBOOK FOR REPORTING RESULTS OF CANCER TREATMENT (1979). *WHO Offset Publication* no. 48. World Health Organization, Geneva.